# A retrospective analysis of tumor infiltrating lymphocytes in head and neck squamous cell carcinoma patients treated with nivolumab

**DOI:** 10.1038/s41598-022-27237-0

**Published:** 2022-12-29

**Authors:** Kiyomi Kuba, Hitoshi Inoue, Satoko Matsumura, Yuichiro Enoki, Yasunao Kogashiwa, Yasuhiro Ebihara, Mitsuhiko Nakahira, Tomoko Yamazaki, Masanari Yasuda, Kyoichi Kaira, Hiroshi Kagamu, Masashi Sugasawa

**Affiliations:** 1 Department of Head and Neck Surgery and Otolaryngology, Saitama Medical University International Medical Center, Hidaka, Saitama Japan; 2 Department of Diagnostic Pathology, Saitama Medical University International Medical Center, Hidaka, Saitama Japan; 3 Department of Respiratory Medicine, Saitama Medical University International Medical Center, Hidaka, Saitama Japan

**Keywords:** Cancer microenvironment, Cancer therapy, Head and neck cancer, Tumour immunology, Cancer, Oncology, Cancer

## Abstract

Nivolumab, an immune checkpoint inhibitor is the first-line therapy for platinum-resistant recurrent/metastatic head and neck cancer, and highly effective for some patients. However, no factors have been identified that could predict response or prognosis after nivolumab administration. We retrospectively investigated the association between tumor infiltrating lymphocytes (TILs) of initial pathology and prognosis in patients treated with nivolumab. Twenty-eight patients with human papilloma virus and Epstein–Barr virus unrelated head and neck squamous cell carcinoma were enrolled. CD8^+^cells, FoxP3^+^cells and FoxP3^−^CD4^+^cells in the tumoral and peritumoral stromal area and PD-L1 were measured. In result, FoxP3^−^CD4^+^TIL, FoxP3^+^TIL, and CD8^+^TIL were not correlated with survival in either intratumoral and stromal area. In univariate analysis, objective response was significant prognostic factor both in progression-free survival and overall survival (*p* = 0.01, 0.006, respectively). PD-L1 was also significant prognostic factor both in progression-free survival and overall survival (*p* = 0.01, 0.01, respectively). ECOG Performance status was a significant prognostic factor in overall survival (*p* = 0.0009). In the combined analysis of stromal CD8^+^TIL and PD-L1, PD-L1 positive with high stromal CD8^+^TIL subgroups had a better prognosis than PD-L1 negative with low stromal CD8^+^TIL subgroups in progression-free survival (*p* = 0.006). Although these results require a further investigation, PD-L1 and ECOG Performance status and the combination of stromal CD8^+^TIL and PD-L1 positivity have potential as useful prognostic markers in patients of virus unrelated head and neck squamous cell carcinoma treated with nivolumab.

## Introduction

Immune checkpoint inhibitors (ICIs) are often used as first-line treatment for platinum-resistant recurrent/metastatic head and neck squamous cell carcinoma (HNSCC), but the low response rate to ICIs is a clinical problem. The response rate to nivolumab, the first monoclonal antibody developed against human programmed death-1 (PD-1), was 13% in the Checkmate 141 trial, and that of pembrolizumab, another PD-1 antibody, used with or without chemotherapy, was approximately 35–40% in the Keynote 048 trial^1,2^. Programmed death-ligand 1 (PD-L1) expression in tumor cells, combined positive score (CPS) which uses the expression rate of PD-L1 in tumor cells and immune cells, tumor mutational burden, tissue microenvironment including tumor-infiltrating lymphocytes (TILs), and microsatellite instability-high have been reported as possible predictive factors, but a consensus on predictors of response to ICIs has not been established except for the CPS of the patients treated with pembrolizumab^3–5^. Because TILs are directly responsible for antitumor immunity, it is critical to elucidate the impact of TILs on the effect of ICIs. Although several reports have shown that TILs change dynamically over the course of treatment, the original TIL status has been shown to correlate with prognosis in many carcinomas^6^. This is no exception in HNSCC patients, and two recent meta-analyses of TILs in HNSCC patients have found that TILs, especially CD8^+^TIL, are associated with prognosis^7,8^. However, the evidence on the correlation between TIL status and nivolumab efficacy in head and neck cancer is not mature. This is because nivolumab is indicated for the treatment of cisplatin-resistant recurrent metastatic disease in head and neck cancer, and therefore, tissue evaluation prior to drug administration is not routinely performed.

As mentioned above, a definitive assessment of this issue cannot be concluded from the original pathological specimen because the TIL status of head and neck cancer patients is altered by treatment and differs from the original status^9^. Furthermore, very few studies have answered the clinical question of whether the original TIL status correlates with prognosis in actual ICI-treated cases. Li et al. reported in their meta-analysis the association between CD8^+^TIL and better clinical outcomes in ICI-treated patients with several cancers including melanoma and non-small cell lung cancer^6^. However, in this analysis, HNSCC cases were only one study, and they had treated with Durvalumab and Tremelimumab, which are not standard treatments. Deng et al. reported that CD8^+^TIL in the primary surgical specimens were a significant favorable prognostic factor in patients with ICI-treated bladder cancer^10^. On the other hand, there are no reports investigating TILs of original specimens in ICI-treated patients with head and neck cancer. The aim of this study was to investigate the correlation between clinicopathological features including original TIL status and progression-free survival (PFS) rate and overall survival (OS) rate in HNSCC patients treated with nivolumab.

## Result

### Patients and clinicopathological characteristics

**Figure 1:**
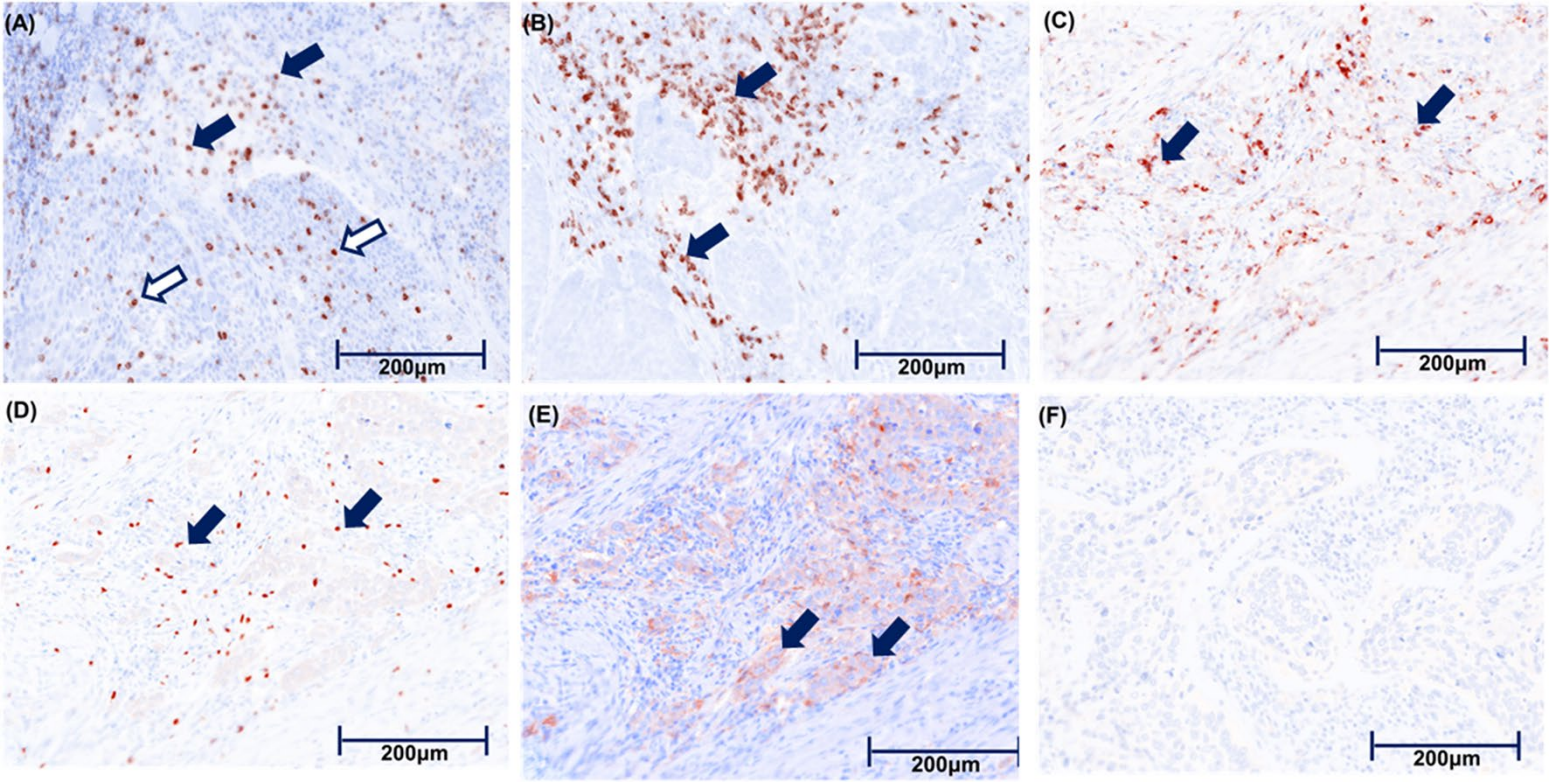
Representative pathological view of immunohistochemistry. (**A**) tumoral (white arrow) and stromal (black arrow) CD8^+^TIL. (**B**) stromal CD8^+^TIL (black arrow). (**C**) stromal CD4^+^TIL (black arrow). (**D**) stromalFoxP3^+^TIL (black arrow). FoxP3^−^CD4^+^Tcell was calculated by excluding CD4^+^FoxP3^+^Tcell from CD4^+^TCell. (**E**) PD-L1 positive case. High PD-L1 expression in tumor cells (black arrow). (**F**) PD-L1 negative case.

Twenty-eight virus-unrelated HNSCC patients treated with nivolumab at our hospital were enrolled. The median observation period was 11 months (2.8–33.3 months). The baseline characteristics of these patients are shown in Table 1. The cut-off value of TILs is shown in Supplementary Table S1. Representative TIL IHC staining is shown in Fig. 1.

**Table 1:** Baseline characteristics of patients.

Characteristics	(N = 28)
Median age, years (range)	66 (36–74)
**Gender, n (%)**	
Male/female	20 (71.4)/8 (28.6)
**Primary site, n (%)**	
Oral cavity	8 (28.6)
Nasopharynx	1 (3.6)
Oropharynx	7 (25.0)
Hypopharynx	9 (32.1)
Larynx	3 (10.7)
**Primary therapy, n (%)**	
Surgery	4 (14.3)
Chemoradiotherapy	6 (21.4)
Surgery and chemoradiotherapy	7 (25.0)
Surgery and radiotherapy	5 (17.9)
Radiotherapy	1 (3.6)
Chemotherapy	5 (17.9)
**Target lesion, n (%)**	
Locoregional	10 (35.7)
Distant metastasis	10 (35.7)
Both	8 (28.6)
**Regimen line of ICIs, n (%)**	
1st/2nd/3rd or more	13 (46.4)/9(32.1)/6(21.4)
**Prior cetuximab therapy, n (%)**	
Yes/no	11 (39.3)/17 (60.7)
**ECOG performance status, n (%)**	
0/1/2	12 (42.9)/13 (46.4)/3 (10.7)
**Specimen, n (%)**	
Surgical resection/Biopsy	18 (64.3)/10 (35.7)

### Correlating clinicopathological characteristics with clinical response

In this study, 3 cases (10.7%) were complete responses, and 7 cases (25.0%) were partial responses, 6 cases (21.4%) were stable disease, and 12 cases (42.9%) were progressive disease. The clinical responses in each variable are shown in Table 2. We created a combined PD-L1 and intratumoral or stromal CD8^+^TIL classification, classifying patients with both PD-L1 positive and high CD8^+^TIL expression (double positive) as class 1, those with PD-L1 negative and low CD8^+^TIL expression(double negative) as class 3, and those with either expression as class 2. There was no significant correlation between clinicopathologic factors and clinical response.

**Table 2:** Relationship between clinicopathological characteristics and objective response.

Characteristics		Responders	Non-responders	p-value
Age (divided by median)	< 66	5	10	1.00
	≥ 66	5	8	
ECOG PS	0	5	7	0.85
	1	4	9	
	2	1	2	
Prior cetuximab therapy	Yes	4	7	1.00
	No	6	11	
Target lesion (local or distant metastasis)	Local	3	7	0.70
	Distant	7	11	
Regimen line	1st	4	9	0.70
	2nd or more	6	9	
PD-L1	< 1%	2	8	0.24
	≥ 1%	8	10	
Tumoral FoxP3^−^CD4^+^TIL	Low	5	9	1.00
	High	5	9	
Tumoral CD8^+^TIL	Low	5	9	1.00
	High	5	9	
Tumoral FoxP3^+^TIL	Low	5	9	1.00
	High	5	9	
Stromal FoxP3^−^CD4^+^TIL	Low	5	9	1.00
	High	5	9	
Stromal CD8^+^TIL	Low	4	9	0.70
	High	6	7	
Stromal FoxP3^+^TIL	Low	6	8	0.69
	High	4	10	
Combined index of PD-L1 and tumoral CD8^+^TIL	1	1	5	0.68
	2	5	7	
	3	4	6	
Combined index of PD-L1 and stromal CD8^+^TIL	1	1	4	0.58
	2	4	9	
	3	5	5	

Patients were classified as responders or non-responders according to objective response to nivolumab. Responders were those who had complete or partial remission as their best response, and non-responders were those with stable or progressive disease.

### Correlating clinicopathological characteristics with survival

**Figure 2:**
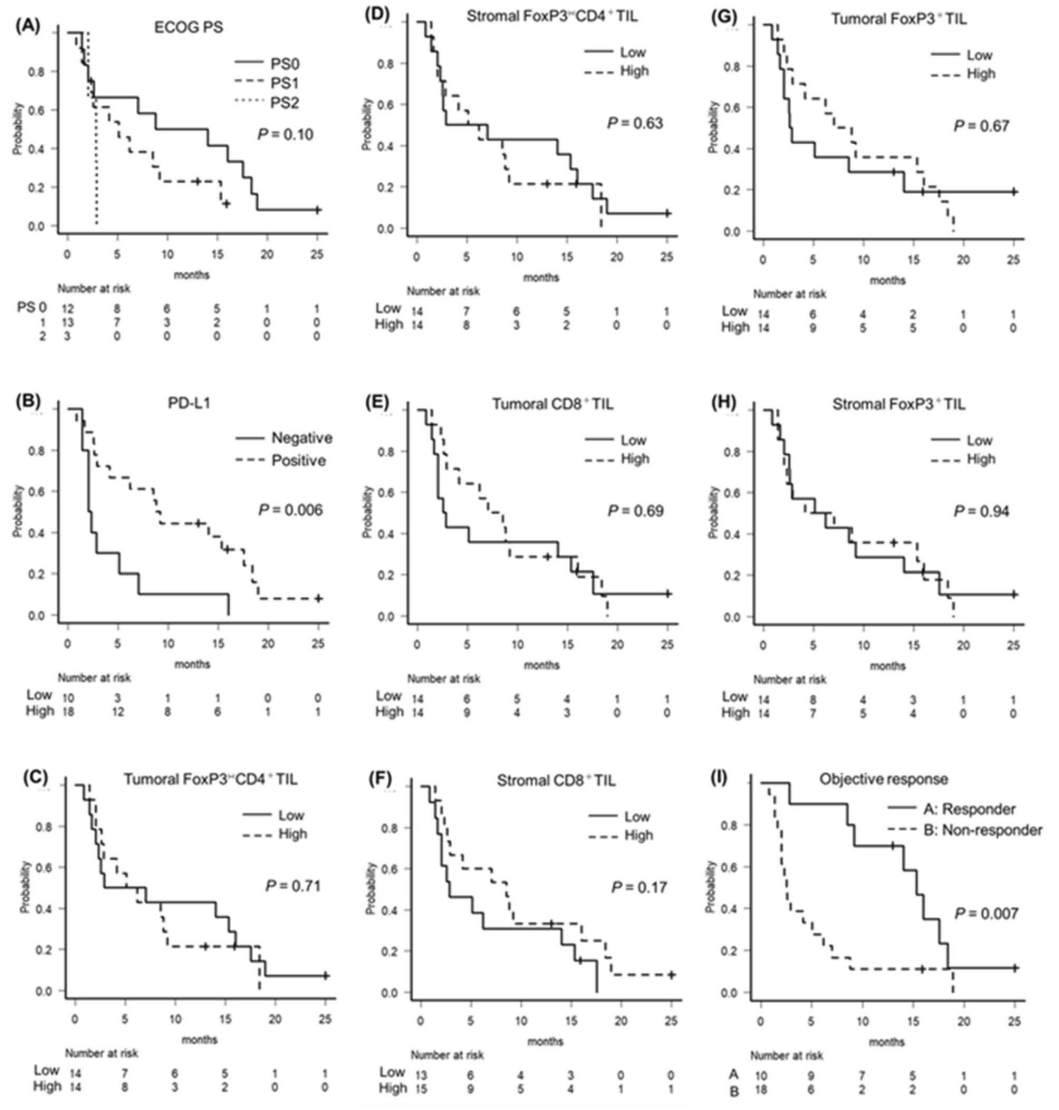
Kaplan–Meier curves for PFS of each type. (**A**) Survival curve of ECOG PS. (**B**) PD-L1. (**C**) tumoral FoxP3^−^CD4^+^TIL. (**D**) stromal FoxP3^−^CD4^+^TIL. (**E**) tumoral CD8^+^TIL. (**F**) stromal CD8^+^TIL. (**G**) tumoral FoxP3^+^TIL. (**H**) stromal FoxP3^+^TIL. (**I**) Objective response.

**Figure 3:**
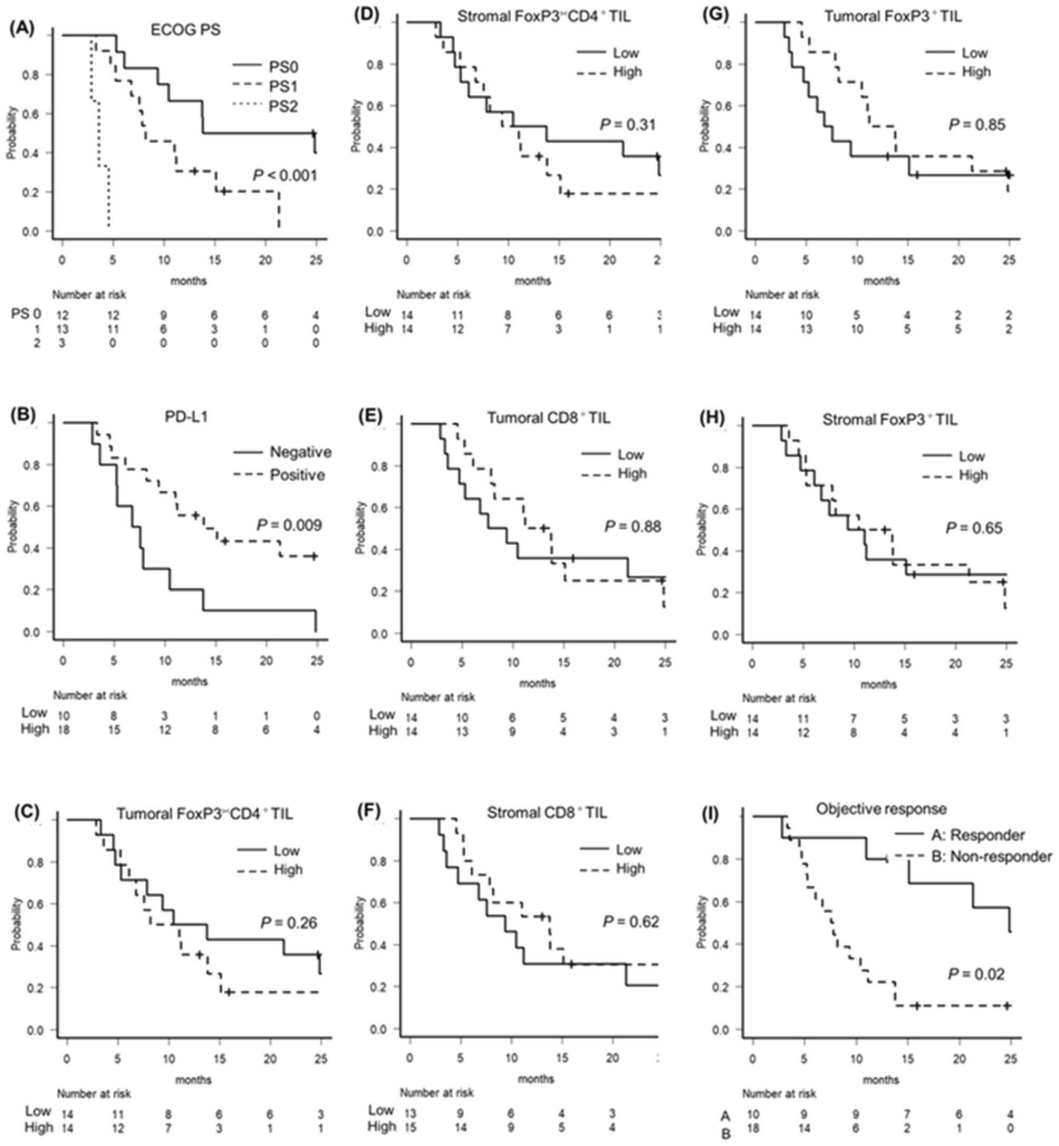
Kaplan–Meier curves for OS of each type. (**A**) Survival curve of ECOG PS. (**B**) PD-L1. (**C**) tumoral FoxP3^−^CD4^+^TIL. (**D**) stromal FoxP3^−^CD4^+^TIL. (**E**) tumoral CD8^+^TIL. (**F**) stromal CD8^+^TIL. (**G**) tumoral FoxP3^+^TIL. (**H**) stromal FoxP3^+^TIL. (**I**) Objective response.

**Figure 4:**
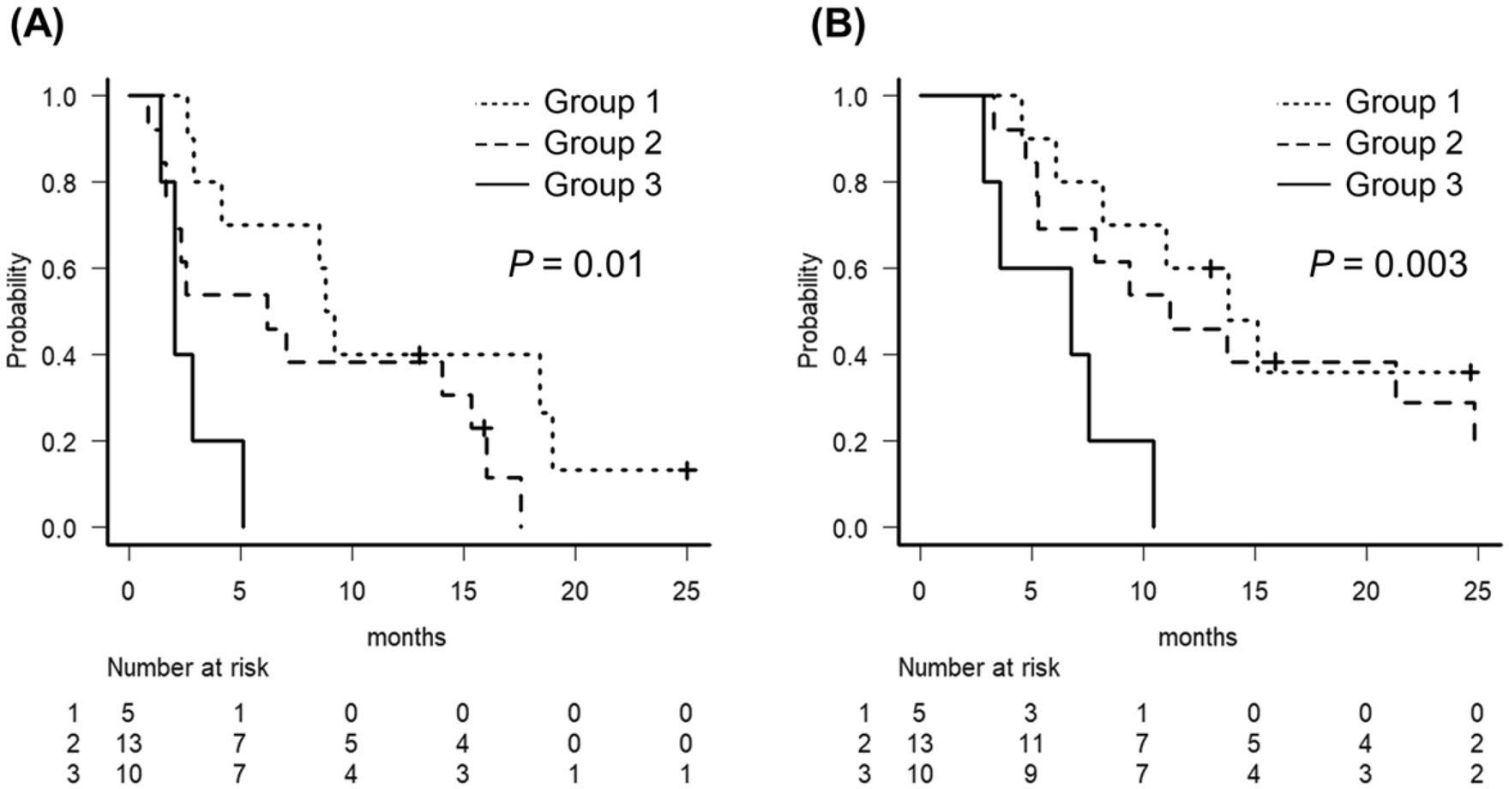
Kaplan–Meier curves of stromal CD8^+^TIL combined with PD-L1. High stromal CD8^+^TIL and PD-L1 positive subgroup were classified as class1 1, Low stromal CD8^+^TIL and PD-L1 negative group were classified as class 3, the other staining group were classified as class2 2. (**A**) PFS curve. (**B**) OS curve.

The median OS time for all cases was 11 months, with a 1-year OS rate of 42.9%. The median PFS time was 5.7 months. Table 3 shows the results of the univariate analysis of each parameter for PFS and OS. Responders had significantly better prognosis for both PFS and OS than non-responders (p = 0.01 and 0.006, respectively). The PD-L1 positive group had significantly better prognosis for both PFS and OS (p = 0.006 and 0.009, respectively), and low score of ECOG Performance Status (PS) had significantly better prognosis for OS (p < 0.001). Although each TILs alone did not correlate with survival, the combined analysis of PD-L1 and CD8^+^TIL was significant better prognostic factor for both PFS and OS (p = 0.009 and 0.02, respectively). The survival curves for each characteristic are shown in Figs. 2 and 3, and the survival curves for the combined analysis of stromal CD8^+^TIL and PD-L1 are shown in Fig. 4. The class 1 subgroup (high stromal CD8^+^TIL and PD-L1 positive) showed the highest PFS and OS. Conversely, the class 3 subgroup (low stromal CD8^+^TIL and PD-L1 negative) showed the worst PFS and OS. The survival curves for other characteristics can be found as Supplementary Fig. S1. The data obtained by analyzing TILs without separating the intratumor and stroma are shown in Supplementary Tables S2 and S3 and Supplementary Fig. S2 and S3. Survival curves for the combined analysis of total CD8^+^TIL and PD-L1 showed similar results as that of stromal CD8^+^TIL.

**Table 3:** Univariate analysis of patients’ PFS and OS.

Variables	PFS		OS	
	HR (95% CI)	*P*	HR (95% CI)	*P*
Age	1.02 (0.98–1.07)	0.19	1.02 (0.97–1.07)	0.35
ECOG performance status (0/1/2)	2.01 (1.01–4.03)	0.04	4.73 (1.87–11.9)	0.0009
Cetuximab treatment (yes vs no)	1.16 (0.49–2.71)	0.72	0.77 (0.33–1.81)	0.56
Regimen line (1st vs 2nd or more)	0.74 (0.33–1.65)	0.47	0.70 (0.30–1.63)	0.41
Target lesion (local vs distant)	1.05 (0.44–2.49)	0.90	0.87 (0.37–2.07)	0.76
Objective response (responders vs non-responders)	0.31 (0.13–0.76)	0.01	0.20 (0.06–0.63)	0.006
PD-L1 (< 1% vs ≥ 1%)	0.31 (0.13–0.76)	0.01	0.32 (0.13–0.79)	0.01
Tumoral FoxP3^−^CD4^+^TIL (high vs low)	1.16 (0.51–2.64)	0.72	1.63 (0.68–3.86)	0.26
Tumoral CD8^+^TIL (high vs low)	0.85 (0.38–1.88)	0.69	1.06 (0.45–2.50)	0.88
Tumoral FoxP3^+^TIL (high vs low)	0.84 (0.37–1.88)	0.68	0.92 (0.40–2.13)	0.85
Stromal FoxP3^−^CD4^+^TIL (high vs low)	1.21 (0.53–2.73)	0.63	1.55 (0.65–3.68)	0.32
Stromal CD8^+^TIL (high vs low)	0.56 (0.24–1.29)	0.17	0.81 (0.35–1.85)	0.62
Stromal FoxP3^+^TIL (high vs low)	1.02 (0.46–2.25)	0.94	1.21 (0.51–2.82)	0.65
Combined index of PD-L1 and tumoral CD8^+^TIL (1/2/3)	0.53 (0.28–1.00)	0.05	0.57 (0.29–1.13)	0.11
Combined index of PD-L1 and stromal CD8^+^TIL (1/2/3)	0.40 (0.20–0.77)	0.006	0.48 (0.24–0.95)	0.03

### Multivariate analysis

To avoid similarity of variables, we performed two multivariate analyses, one with PD-L1 as a variable and the other with a combined index of PD-L1 and CD8^+^TIL (Table 4). Objective response was an independent prognostic factor for both PFS and OS in both settings (p = 0.01, 0.02, respectively). PD-L1 was also an independent prognostic factor for both PFS and OS (p = 0.01, 0.01, respectively). The combined index of stromal CD8^+^TIL and PD-L1 was an independent factor for PFS (p = 0.03). ECOG PS was an independent factor for OS in both settings. These results were similar with using combined index of total CD8^+^TIL and PD-L1 (see Supplementary Table S4).

**Table 4:** Multivariate analysis of patients’ PFS and OS.

Variables	PFS		OS	
	HR (95% CI)	*P*	HR (95% CI)	*P*
**(A)**				
Objective response (responders vs non-responders)	0.33 (0.13–0.82)	0.01	0.25 (0.07–0.85)	0.02
ECOG Performance status (0/1/2)	1.94 (0.97–3.88)	0.05	5.69 (2.13–15.2)	0.0005
PD-L1 (< 1% vs ≥ 1%)	0.31(0.12–0.79)	0.01	0.27(0.09–0.78)	0.01
**(B)**				
Objective response (responders vs non-responders)	0.34 (0.14–0.84)	0.01	0.23 (0.06–0.79)	0.01
ECOG Performance status (0/1/2)	1.50 (0.69–3.23)	0.29	4.27 (1.66–10.9)	0.002
Combined index of PD-L1 and stromal CD8^+^TIL (1/2/3)	0.48 (0.24–0.95)	0.03	0.70 (0.34–1.41)	0.31

(A) Multivariate analysis using PD-L1. (B) Multivariate analysis using combined index of PD-L1 and stromal CD8^+^TIL.

## Discussion

Numerous reports, including HNSCC, have described the correlation between TILs and prognosis of cancer patients, of which CD8^+^TIL is considered the most reliable prognostic factor. Spector et al. reported that higher CD8^+^TIL counts and higher TIL weighted sum scores (including CD4^+^TIL, CD8^+^TIL, and FoxP3^+^TIL) were associated with OS improvements in 464 patients with HNSCC^11^. Meulenaere et al. also reported that CD8^+^TIL was an independent prognostic marker in 100 patients diagnosed with oropharyngeal squamous cell carcinoma^12^. In recent years, several systematic reviews and meta-analyses have reported the importance of TILs in HNSCC. Ruiter et al. reported in the meta-analysis of 16 studies that CD3^+^TIL and CD8^+^TIL were associated with better OS and disease-specific survival (DFS)^7^. Borsetto et al. meta-analyzed 28 studies and reported that high CD4^+^TIL and CD8^+^TIL were associated with a better prognosis for oropharyngeal cancer with and without human papilloma virus (HPV), and high CD8^+^TIL was associated with a prognosis for hypopharyngeal cancer^8^. However, these studies did not include patients actually treated with ICIs, and it is unclear whether CD8^+^TIL is associated with the therapeutic effect of ICIs. In our study, there was no correlation between each TIL and prognosis, including CD8-positive cells, suggesting that each TIL alone does not affect survival after ICI. Of course, our result does not negate the findings of previous meta-analyses because we limit the observation period to after the start of nivolumab treatment. On the other hand, Hanna et al. evaluated TILs in 42 actual ICI-treated virus-negative HNSCC patients with fresh specimens collected before ICI administration and reported that CD8^+^TIL levels were higher in responders compared to non-responders^13^. However, no other reports have evaluated TILs in head and neck cancer patients treated with ICI, and no meta-analysis exists. Future validation in a large cohort using fresh specimens before ICI treatment is needed.

FoxP3 is a marker of immunosuppressive regulatory T cells (Treg)^14^. Therefore, immune response to ICIs is predicted to be poor in the presence of FoxP3 positive cells. However, the significance of FoxP3^+^TIL in HNSCC patients is not clear. In a meta-analysis of 15,512 cancer patients in 76 studies, high FoxP3^+^Treg infiltration was associated with shorter OS in many solid tumors but with improved OS in colorectal, esophageal, and HNSCC^15^. Similarly, Seminerio et al. reported a positive correlation between FoxP3^+^TIL and OS in 205 patients with HNSCC^16^. However, these studies have several issues, including short observation periods and not evaluating other TILs, such as CD8^+^TIL. Spector et al. analyzed TILs in 464 patients with HNSCC, and FoxP3^+^TIL alone was not a significant factor for prolonged OS and DFS, whereas high CD8^+^TIL infiltration correlated with significantly better prognosis^11^. In a meta-analysis of Treg in HNSCC, Cho et al. reported that circulating Tregs in peripheral blood correlated with high survival rates, with no significant difference in Tregs in tissues^17^. As mentioned above, there is no established evidence for the prognostic value of FoxP3^+^TIL in HNSCC. It is speculated that FoxP3^+^TIL is not a direct prognostic factor but an indirect factor due to a positive correlation with TILs such as CD8^+^TIL^18^. Our study also showed no correlation between Foxp3^+^TIL and survival, and therefore the original FoxP3^+^TIL did not correlate with prognosis with or without ICI treatment.

Regarding PD-L1 expression and prognosis, in our study, PD-L1 positive was related to a significant better OS and PFS both in univariate and multivariate analysis. Yang et al. reported no significance between PD-L1 positivity and prognosis in a meta-analysis of 3105 HNSCC cases^19^. Li et al. conducted a meta-analysis of 17 articles and reported that PD-L1 expression level did not predict OS in HNSCC and was a poor prognostic factor in the Asian subgroup^20^. However, these two meta-analyses included few cases treated with ICIs. In a meta-analysis of ICI-treated cases, Liu et al. reported a correlation between PD-L1 positivity and good response in solid tumors^21^. Furthermore, in head and neck cancer, Huang et al. reported a favorable correlation between PD-L1 positivity and OS and objective response rate in a meta-analysis of 1663 ICI-treated HNSCC patients in 11 studies^22^. The survival curves of our cases showed that PD-L1-positive patients had a significantly better prognosis than PD-L1-negative patients, consistent with these results. Furthermore, PD-L1 was an independent factor in multivariate analysis. Therefore, PD-L1 appears to be the most promising prognostic factor in nivolumab-treated patients with virus-unrelated head and neck cancer.

However, it is difficult to completely predict the efficacy of nivolumab based on PD-L1 expression alone, since there are cases in which PD-L1 is positive without ICI efficacy. The PD-1/PD-L1 pathway inhibits the activation of T cell immunity, achieving tumor immune escape and blocking cancer immunity cycles. However, the mechanism underlying tumor immunosuppression involves many factors other than the PD-1/PD-L1 pathway, such as CTLA4 and IDO-1^23^. Therefore, it is difficult to select all responders based on only PD-L1 expression. On the other hand, when the PD-1/PD-L1 pathway is the main cause of tumor immunosuppression accompanied by CD8^+^TIL infiltration, the correlation between PD-L1 positivity and therapeutic efficacy of ICI is estimated to be high. Teng et al. classified the expression forms of TILs and PD-L1 into four categories^24^. Type 1 tumor is characterized by PD-L1 positivity with high TILs (adaptive immune resistance); the benefit of ICI is most likely to be obtained. Type 2 tumor has PD-L1 negativity with low TILs (immune ignorance); the prognosis of this tumor type is very poor. Type 3 tumor is characterized by PD-L1 positivity with low TILs (intrinsic induction), with difficulty in obtaining the benefit of ICI even when PD-L1 expression is present. Type 4 tumor shows PD-L1 negativity with high TILs (immune tolerance), which seems to be predominantly influenced by immunosuppressive pathways other than PD-1/PD-L1. Canteli et al. analyzed 372 surgically treated HPV-negative HNSCC patients and reported better DFS in cases with Type 1^18^. Hu et al. also reported that the combination of PD-L1 and high CD8 expression was a prognostic factor and associated with improved OS in 111 patients with hypopharyngeal SCC^25^. Although PD-L1 is likely the main prognostic factor, but its combination with stromal CD8^+^TILs may further increase its detectability. In the present study, the PFS curves showed the best for the class 1 subgroup, followed by the class 2 subgroup, and the class 3 subgroup showed the worst prognosis. Our result suggests that nivolumab may be less effective in patients with negative PD-L1 and low CD8^+^TIL expression, which is consistent with the report of Teng et al. However, the detectability of this study is low due to the small number of cases, and validation in a larger cohort would be desirable. Moreover, as mentioned above, the present study was based on the original specimen, and the direct relationship between the effect of ICI and pathological factors is uncertain. In order to accurately assess these factors, evaluation using fresh specimens before ICI administration would be desirable.

In this study, stromal CD8^+^TIL was useful for combined factor rather than intratumoral CD8^+^TIL. The area where TILs have been most researched is breast cancer, and guidelines for TIL analysis have been proposed by the International Immuno-Oncology Biomarkers Working Group^26^. In this context, the initial hypothesis was that intratumoral TILs, which interact directly with cancer cells, were more relevant to cancer immunity, but most current studies have found that stromal TILs are a more reproducible parameter in predicting therapeutic response than intratumoral TILs. This is because intratumoral TILs are less frequently expressed compared to stromal TILs, making their recognition and scoring difficult. In our study, intratumoral CD8^+^TIL and stromal CD8^+^TIL show a strong correlation (r = 0.78, p < 0.0001), and stromal CD8^+^TIL were also more frequently expressed, and the more accurate scoring of stromal CD8^+^TIL may have contributed to the significance of the results. However, there have been reports that intratumoral TILs are useful in head and neck cancer, and the impact of intratumoral TILs should continue to be investigated^27^.

Furthermore, in our study, there was a significant correlation between ECOG PS and prognosis, and no correlation between age, site of recurrence/metastasis, or smoking history. ECOG PS has been shown to correlate with better survival after ICI treatment in other solid tumors such as lung cancer and malignant melanoma^28,29^. In head and neck cancer, Singh et al. and Hanai et al. reported a positive correlation between ECOG PS and survival in patients treated with nivolumab^30,31^. Based on these reports and our results, the prognosis for patients with a ECOG PS score 2 or higher is extremely poor, and the indication for ICI treatment should be carefully considered.

Our study has some limitations. First, due to the small number of cases it is difficult to conclude these results definitively and these results should be validated in larger cohorts in the future. Second, this study was not evaluated using specimens just prior to ICI administration. Because TILs are altered by conventional therapy, specimens obtained immediately prior to ICI administration should be used to evaluate TILs as a predictor of ICI efficacy. Third, it did not include many other factors that play important roles in the cancer microenvironment, such as B cells, macrophages, and fibroblasts. Thus, our results were obtained from a limited number of immune factors. Although PD-L1 and combined analysis of PD-L1 and CD8^+^TIL are certainly an important factors based on existing reports, it should be considered a surrogate marker reflecting a complex immune mechanism.

## Conclusion

PD-L1 and ECOG PS were useful prognostic factors for virus negative HNSCC patients treated with nivolumab. Although TILs have no direct impact on prognosis, combination of PD-L1 and stromal CD8^+^TIL may be useful in predicting prognosis. Further evaluation with fresh specimens prior to ICI treatment in a large cohort is needed.

## Material and methods

### Patients and specimens

Patients treated with nivolumab between September 2017 and January 2021 at our hospital were enrolled in this study. All patients had recurrent or metastatic HNSCC and prior-treated with cisplatin contained chemotherapy. Patients were included if their ECOG PS was 0–2, they were histopathologically diagnosed as having squamous cell carcinoma and initial samples of primary lesion (biopsy or surgical specimen) could be assessed, and they could receive more than 3 times of administration of nivolumab and they could give a valid informed consent. Patients diagnosed with HPV-related oropharyngeal carcinoma and Epstein–Barr virus (EBV)-related nasopharyngeal carcinoma were excluded because of their favorable prognosis. In addition, cases in which efficacy could not be evaluated due to early death or withdrawal were also excluded. The patients received 3 mg/kg nivolumab intravenously every 2 weeks before August 2018 and 240 mg/body after September 2018 until disease progression or withdrawal from treatment. Baseline characteristics and clinical course data were retrospectively collected from electronic medical records. Smoking history was excluded from the analysis because the majority of the patients were smokers (25 smokers and 3 non-smokers), and proportional hazard property was not valid. Tumor responses were assessed by computed tomography or magnetic resonance imaging at baseline and every 8–12 weeks after treatment initiation. Objective response was evaluated using RECIST version 1.1. Based on RECIST criteria, the patients were classified as responders (complete remission or partial remission) and non-responders (stable disease or progressive disease). Initial surgical tissue, or biopsy specimen in absence of surgical tissue, were used for pathological diagnosis.

### Immunohistochemistry and data analysis

For evaluation of TILs, FoxP3^−^CD4^+^TIL as helper T cells, CD8^+^TIL as cytotoxic T cells, and FoxP3^+^ TIL as regulatory T cells were investigated. Formalin-fixed paraffin-embedded tissue specimens were cut into 5 µm-thick sections and prepared for immunohistochemistry (IHC) and multiplex immunofluorescence (IF) imaging. The sections were deparaffinized and rehydrated with xylene and ethanol for multiplex IHC staining. All slides were sequentially treated with 0.3% hydrogen peroxide in methanol for 30 min to block endogenous peroxidase activity. Next, all sections were autoclaved in 10 mmol L^−1^ sodium citrate buffer (pH 6.0) for 20 min, microwaved at 98 °C for 15 min to expose antigens, and cooled for 30 min. All sections were then rinsed in 0.05 M tris-buffered saline, containing 0.1% tween 20, and incubated with mouse monoclonal CD4 antibody (Leica Biosystems, Buffalo Grove, IL, USA, clone 4B12, 1:100, high pH retrieval), mouse monoclonal CD8 antibody (DAKO, Carpinteria, CA, USA, clone C8/144B 1:150, high pH retrieval), mouse monoclonal FOXP3 antibody (Abcam, Cambridge, UK, clone 236A/E7, 1:50, pH6 retrieval), rabbit monoclonal PD-L1 (Abcam, Cambridge, UK, clone 28-8, 1:100, pH6 retrieval), and mouse monoclonal pan-cytokeratin antibody (Abcam, Cambridge, UK, clone AE1/AE3 1:100, pH6 retrieval). Immunofluorescence was visualized using the OPAL Multiplex IHC kit (Akoya Biosciences, Marlborough, MA, USA). All slides were imaged on the Mantra 2 Quantitative Pathology Workstation (Akoya Biosciences). Separation by color, segmentation of tissue and cell, and phenotyping of all cells was performed using inForm Software v2.5.1 (Akoya Biosciences) to extract image data. The slides were evaluated for the presence of TILs within the intratumoral and stromal regions. Multiplex IHC staining and data analyses were performed according to earlier reports^32^. An algorithm of image analysis was designed based on pattern recognition of pan-cytokeratin-positive areas (tumor) and pan-cytokeratin-negative areas (peritumoral stroma). Cell segmentation was performed according to all cells counterstained with DAPI. The TIL scoring for distribution was performed on three 20 × scanned images randomly selected from marginal tumor regions with high TIL density in each patient. All tumor cells were recognized by numbering in the analysis, and cells stained for both FoxP3 and CD4 were counted as FoxP3^+^Cells, and cells stained for CD4 and not stained for FoxP3 were counted as FoxP3^−^CD4^+^ cells. For normalization, the total number of cells in the intratumoral and stromal regions was adjusted to 1000. The average value was calculated from the total number of these three regions and used as the TIL value for each selected specimen. The median value was used as the cut-off point to divide high and low expressions, as earlier^33^. PD-L1 expression was assessed by the tumor proportion score, and 1% or more were defined as positive.

### Statistical analysis

The pathological characteristics included FoxP3^−^CD4^+^TIL, CD8^+^TIL, FoxP3^+^TIL, and PD-L1. The high and low expressions of these markers were defined according to cut-off values, as described above. We created a combined PD-L1 and CD8^+^TIL classification, classifying patients with both PD-L1 positive and high CD8^+^TIL expression (double positive) as class 1, those with PD-L1 negative and low CD8^+^TIL expression (double negative) as class 3, and those with either expression as class 2. All TILs in the tumoral and stromal areas were evaluated separately. The patients characteristics included age, ECOG PS (0, 1, 2), Regimen line (1st or 2nd or more); whether nivolumab was given as a first-line drug for residual or recurrent disease, Target lesion (locoregional or distant); whether the target lesion include distant metastasis or only locoregional, and treatment history of cetuximab, and objective response (responders or non-responders). Among these variables, age was evaluated as a continuous variable for univariate analysis or ordinal variable for clinical response with 66-years old as cut off. Regimen line, target lesion, and history of cetuximab treatment, and objective response as nominal variables, and the rest as ordinal variables. PFS and OS were calculated from the first ICI administration date to the date of an event (death or disease progression for PFS and death for OS). Patients were censored if there was no progression during observation for PFS and no death for OS during follow-up. Χ^2^ test was used for categorical variables, and Fisher’s exact test was used for smaller group sizes that are less than five. Spearman rank testing was used to assess correlations between two variables. Kaplan–Meier analysis and log-rank tests were used to estimate survival curves. For univariate, Cox proportional hazard models were used to calculate hazard ratios and 95% confidence intervals, and multivariate analysis was performed using factors that were significant in univariate analysis. We verified the proportional hazard property of the variables by the EZR software, with the null hypothesis that the proportional hazard property is satisfied not being rejectable. All tests were two-tailed, and a P-value of < 0.05 was considered significant. All statistical analyses were performed using EZR software^34^.

### Ethical approval

The study protocol complied with the Declaration of Helsinki and was approved by the institutional review board of Saitama Medical University International Medical Center (approval no. 17-044, 17-262). All patients provided written informed consent prior to enrollment.

## Supplementary Information

Supplementary Information.

## Data Availability

The datasets analyzed for this study can be found in the [figshare] [https://doi.org/10.6084/m9.figshare.21304677].
