# Author Correction: A retrospective analysis of tumor infiltrating lymphocytes in head and neck squamous cell carcinoma patients treated with nivolumab

**DOI:** 10.1038/s41598-023-28598-w

**Published:** 2023-01-26

**Authors:** Kiyomi Kuba, Hitoshi Inoue, Satoko Matsumura, Yuichiro Enoki, Yasunao Kogashiwa, Yasuhiro Ebihara, Mitsuhiko Nakahira, Tomoko Yamazaki, Masanari Yasuda, Kyoichi Kaira, Hiroshi Kagamu, Masashi Sugasawa

**Affiliations:** 1 Department of Head and Neck Surgery and Otolaryngology, Saitama Medical University International Medical Center, Hidaka, Saitama Japan; 2 Department of Diagnostic Pathology, Saitama Medical University International Medical Center, Hidaka, Saitama Japan; 3 Department of Respiratory Medicine, Saitama Medical University International Medical Center, Hidaka, Saitama Japan

Correction to: *Scientific Reports* 10.1038/s41598-022-27237-0, published online 29 December 2022

The original version of this Article contained an error in Table 3, where the values in row “Combined index of PD-L1 and tumoral CD8^+^TIL (1/2/3)” were swapped with the values in row “Combined index of PD-L1 and stromal CD8^+^TIL (1/2/3)” in columns “PFS” and “OS”. The correct and incorrect Tables appear below.

Incorrect:

**Table:** 

Variables	PFS		OS	
	HR (95% Cl)	*P*	HR (95% Cl)	*P*
Combined index of PD-L1 and tumoral CD8^+^TIL (1/2/3)	0.40 (0.20–0.77)	0.006	0.48 (0.24–0.95)	0.03
Combined index of PD-L1 and stromal CD8^+^TIL (1/2/3)	0.53 (0.28–1.00)	0.05	0.57 (0.29–1.13)	0.11

Correct:

**Table:** 

Variables	PFS		OS	
	HR (95% Cl)	*P*	HR (95% Cl)	*P*
Combined index of PD-L1 and tumoral CD8^+^TIL (1/2/3)	0.53 (0.28–1.00)	0.05	0.57 (0.29–1.13)	0.11
Combined index of PD-L1 and stromal CD8^+^TIL (1/2/3)	0.40 (0.20–0.77)	0.006	0.48 (0.24–0.95)	0.03

The original Article has been corrected.

