# Author Correction: A retrospective analysis of tumor infiltrating lymphocytes in head and neck squamous cell carcinoma patients treated with nivolumab

**DOI:** 10.1038/s41598-023-35683-7

**Published:** 2023-05-25

**Authors:** Kiyomi Kuba, Hitoshi Inoue, Satoko Matsumura, Yuichiro Enoki, Yasunao Kogashiwa, Yasuhiro Ebihara, Mitsuhiko Nakahira, Tomoko Yamazaki, Masanari Yasuda, Kyoichi Kaira, Hiroshi Kagamu, Masashi Sugasawa

**Affiliations:** 1 Department of Head and Neck Surgery and Otolaryngology, Saitama Medical University International Medical Center, Hidaka, Saitama Japan; 2 Department of Diagnostic Pathology, Saitama Medical University International Medical Center, Hidaka, Saitama Japan; 3 Department of Respiratory Medicine, Saitama Medical University International Medical Center, Hidaka, Saitama Japan

Correction to: *Scientific Reports* 10.1038/s41598-022-27237-0, published online 29 December 2022

The original version of this Article contained errors.

In the Material and methods section, under the subheading ‘Patients and specimens’,

“Patients treated with nivolumab between September 2016 and January 2021 at our hospital were enrolled in this study.”

now reads:

“Patients treated with nivolumab between September 2017 and January 2021 at our hospital were enrolled in this study.”

In addition, in the Ethical approval section,

“The study protocol complied with the Declaration of Helsinki and was approved by the institutional review board of Saitama Medical University International Medical Center (approval no. 17-044). All patients provided written informed consent prior to enrollment.”

now reads:

“The study protocol complied with the Declaration of Helsinki and was approved by the institutional review board of the Saitama Medical University International Medical Center (approval no. 17-044, 17-262). All patients provided written informed consent prior to enrollment.”

The original Article has been corrected.

