# Supplementary material for: A retrospective analysis of tumor infiltrating lymphocytes in head and neck squamous cell carcinoma patients treated with nivolumab
Source: Sci Rep. 2022 Dec 29;12:22557. doi: 10.1038/s41598-022-27237-0 (PMC9800384; doi:10.1038/s41598-022-27237-0)
Supplement: Supplementary file 1 — Supplementary Information. [file 41598_2022_27237_MOESM1_ESM.docx]

Supplementary Material

# Supplementary Tables

**Supplemental Table S1: Pathological characteristics**

| **Pathological characteristics** | **Median value (range)** | |
| --- | --- | --- |
| Tumoral FoXP3^-^CD4^+^TIL | 0.8 | (0–322) |
| Tumoral CD8^+^TIL | 9,5 | (0–201) |
| Tumoral FoxP3^+^TILs | 12.8 | (0–125) |
| Stromal FoXP3^-^CD4^+^TIL | 6.5 | (0-229) |
| Stromal CD8^+^TIL | 20.0 | (0-270) |
| Stromal FoXP3^-^CD4^+^TIL | 27.5 | (0-202) |

**Supplemental Table S2: Pathological characteristics (total TIL)**

| **Pathological characteristics** | **Median value (range)** | |
| --- | --- | --- |
| FoXP3^-^CD4^+^TIL (tumoral + stromal) | 8.5 | (0–570) |
| CD8^+^TIL (tumoral + stromal) | 37.3 | (0–402) |
| FoxP3^+^TILs (tumoral + stromal) | 39.0 | (0–293) |

**Supplemental Table S3. Univariate analysis of patient's PFS and OS.**

|  | **PFS** | | **OS** | |
| --- | --- | --- | --- | --- |
| **Variables** | **HR (95%CI)** | P | **HR (95%CI)** | P |
| CD4^+^ FoxP3^-^TIL (tumoral + stromal) | 1.21(0.53-2.76) | 0.63 | 1.55(0.65-3.68) | 0.32 |
| CD8^+^TIL (tumoral + stromal) | 0.50(0.21-1.15) | 0.10 | 0.74(0.32–1.69) | 0.47 |
| FoxP3^+^TIL (tumoral + stromal) | 0.91(0.41–2.01) | 0.82 | 1.14(0.48-2.71) | 0.76 |
| combined index of PD-L1 and CD8^+^TIL (tumoral + stromal) | 0.38(0.20–0.74) | 0.004 | 0.48(0.25–0.93) | 0.03 |

**Supplemental Table S4. Multivariate analysis of patient's PFS and OS.**

| **(A)** | **PFS** | | **OS** | |
| --- | --- | --- | --- | --- |
| **Variables** | **HR (95%CI)** | P | **HR (95%CI)** | P |
| Objective response  (responders vs non-responders) | 0.33(0.13-0.82) | 0.01 | 0.25(0.07-0.85) | 0.02 |
| ECOG Performance status (0/1/2) | 1.94(0.97-3.88) | 0.05 | 5.69(2.13-15.2) | 0.0005 |
| PD-L1(<1% vs ≥1%) | 0.31(0.12-0.79) | 0.01 | 0.27(0.09–0.78) | 0.01 |
| **(B)** | **PFS** | | **OS** | |
| **Variables** | **HR (95%CI)** | P | **HR (95%CI)** | P |
| Objective response  (responders vs non-responders) | 0.35(0.14-0.86) | 0.02 | 0.23(0.06-0.79) | 0.01 |
| ECOG Performance status (0/1/2) | 1.49(0.68–3.29) | 0.31 | 4.27(1.65–11.0) | 0.002 |
| combined index of PD-L1 and CD8^+^TIL (tumoral + stromal) (1/2/3) | 0.47(0.23-0.92) | 0.02 | 0.73(0.37-1.42) | 0.35 |

**(A)** Multivariate analysis using PD-L1. **(B)** Multivariate analysis using combined index of PD-L1 and stromal CD8^+^TIL.

# Supplementary Figures


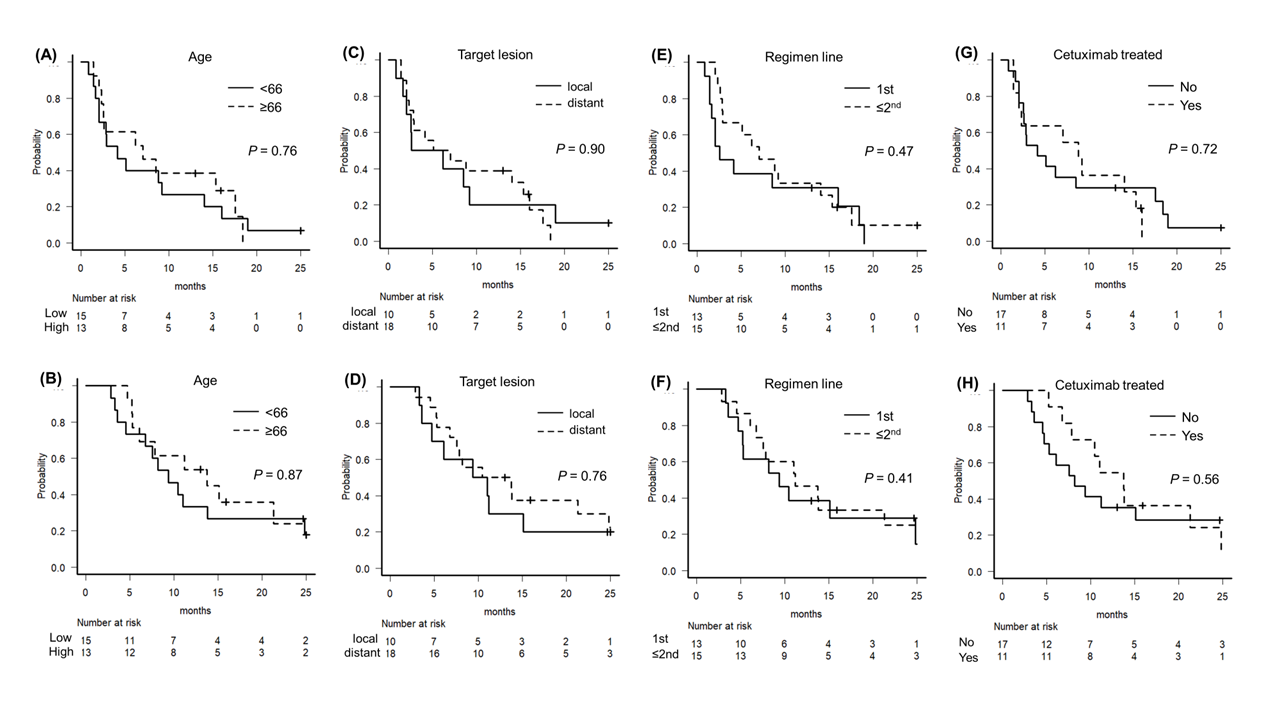


**Supplementary Figure S1.** Kaplan–Meier curves for PFS and OS of each TIL (total means sum of tumoral and stromal cells) type. **(A)** PFS curve of Age. **(B)** OS curve of Age. **(C)** PFS curve of Target lesion (local or distant). **(D)** OS curve of Target lesion (local or distant). **(E)** PFS curve of Regimen line of nivolumab. **(F)** OS curve of Regimen line of nivolumab. **(G)** PFS curve of History of Cetuximab treated. **(H)** OS curve of History of Cetuximab treated.

**
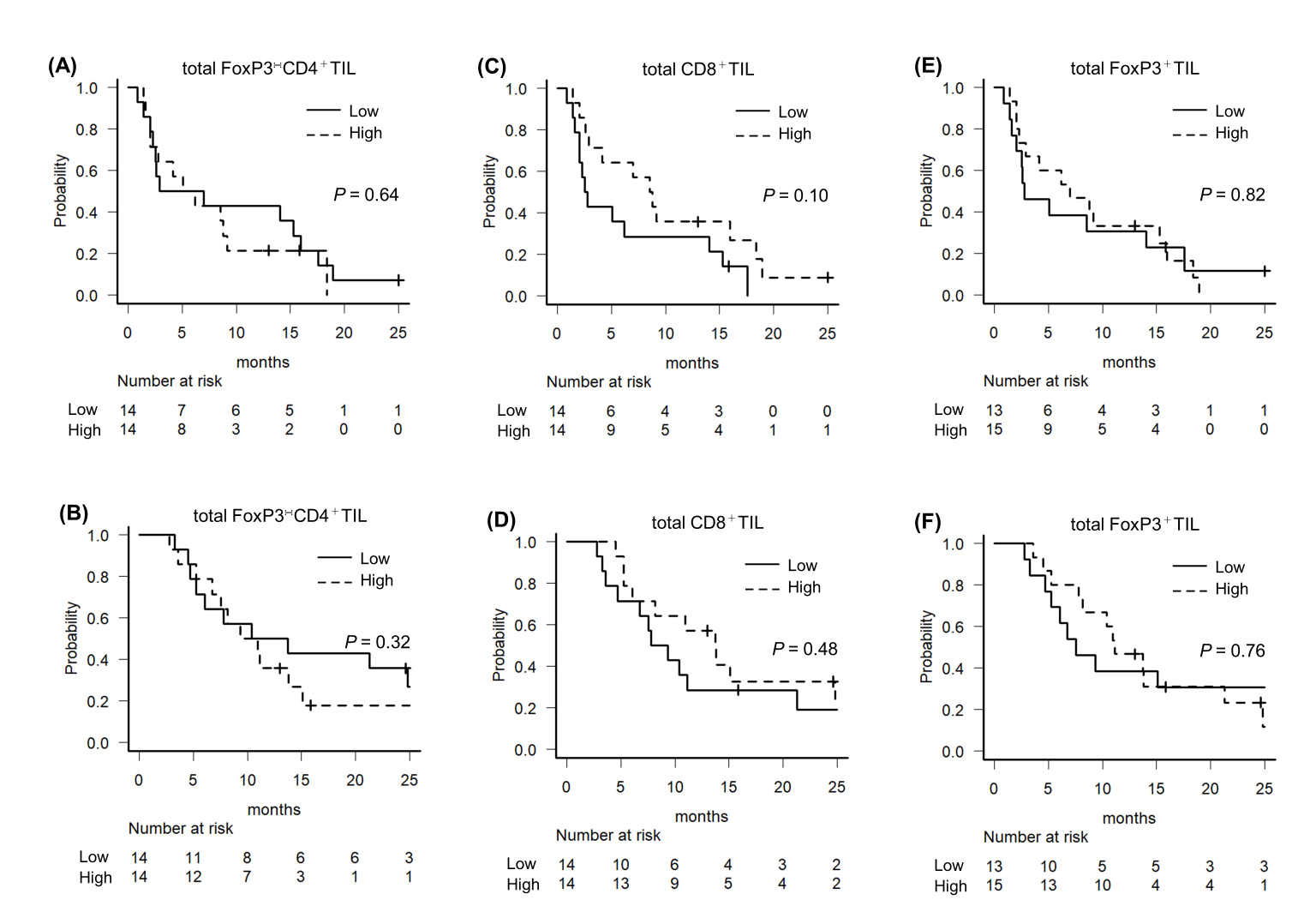
**

**Supplementary Figure S2.** Kaplan–Meier curves for PFS and OS of each TIL (total means sum of tumoral and stromal cells) type. **(A)** PFS curve of total FoxP3^−^CD4^+^TIL. **(B)** OS curve of total FoxP3^−^CD4^+^TIL. **(C)** PFS curve of total CD8^+^TIL. **(D)** OS curve of total CD8^+^TIL. **(E)** PFS curve of total FoxP3^+^TIL. **(F)** OS curve of total FoxP3^+^TIL.


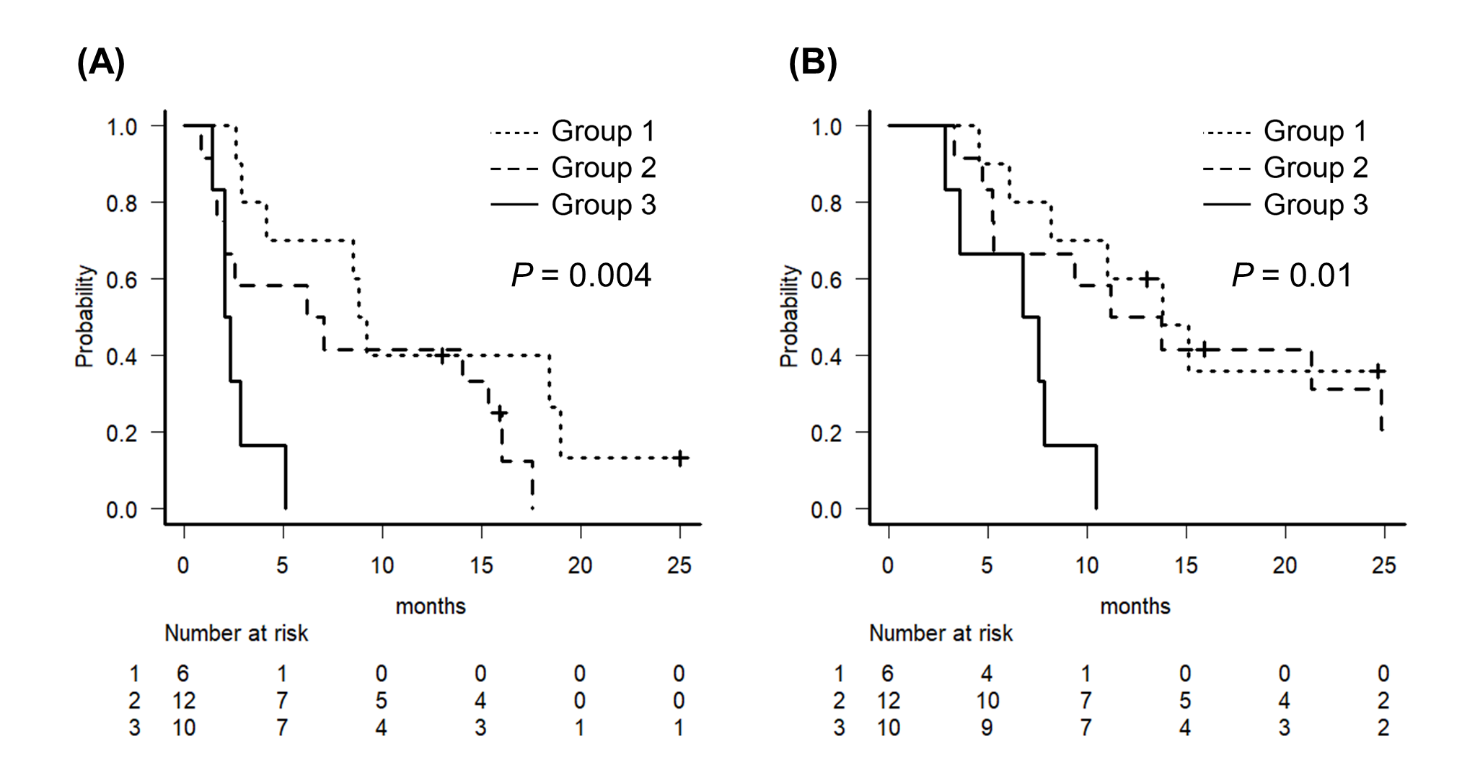


**Supplementary Figure S3.**

Kaplan–Meier curves of total CD8^+^TIL combined with PD-L1. High total CD8^+^TIL and PD-L1 positive subgroup were classified as class 1, low total CD8^+^TIL and PD-L1 negative subgroup were classified as class 3, and the other staining subgroup were classified as class 2. **A**, PFS curve. **B**, OS curve.
